# 2,4-Disubstituted pyridine derivatives are effective against intracellular and biofilm-forming tubercle bacilli

**DOI:** 10.3389/fphar.2022.1004632

**Published:** 2022-11-10

**Authors:** M. Korycka-Machała, M. Kawka, J. Lach, R. Płocińska, A. Bekier, B. Dziadek, A. Brzostek, P. Płociński, D. Strapagiel, M. Szczesio, K. Gobis, J. Dziadek

**Affiliations:** ^1^ Laboratory of Genetics and Physiology of Mycobacterium, Institute of Medical Biology of the Polish Academy of Sciences, Lodz, Poland; ^2^ Department of Molecular Microbiology, Faculty of Biology and Environmental Protection, University of Lodz, Lodz, Poland; ^3^ Biobank Lab, Department of Molecular Biophysics, Faculty of Biology and Environmental Protection, University of Lodz, Lodz, Poland; ^4^ Department of Immunology and Infectious Biology, Faculty of Biology and Environmental Protection, University of Lodz, Lodz, Poland; ^5^ Institute of General and Ecological Chemistry, Faculty of Chemistry, Lodz University of Technology, Poland; ^6^ Department of Organic Chemistry, Medical University of Gdansk, Gdansk, Poland

**Keywords:** pyridine derivatives, tuberculosis, drug resistance, Mtb inhibitors, MmpR5, biofilm

## Abstract

It was recently reported that 4-substituted picolinohydrazonamides carrying hydrophilic cyclic amines, such as morpholine and pyrrolidine, at the end of their thiosemicarbazide chain have potent antimycobacterial activity *in vitro* at concentrations below 1 μg/ml. Here, two selected compounds, 2,4-disubstituted pyridine derivatives **11** and **15**, revealed significant bactericidal activity against *Mycobacterium tuberculosis* localized intracellularly within human macrophages, as well as against biofilm-forming tubercle bacilli. Mutants were selected that were resistant to the investigated compounds at an efficiency similar to that identified in the presence of the first line antituberculosis drug rifampicin. The resistant mutants were viable in the presence of the tested compounds exclusively on solid media. Genome-wide sequencing of the mutants selected in the presence of compound **11** revealed the accumulation of nonsynonymous mutations in the *mmpR5* gene encoding a transcriptional repressor of the MmpS5-MmpL5 efflux pump, whose upregulation has been associated with bedaquiline resistance. The depletion of MmpR5 in wild-type *M. tuberculosis* using CRISPR–Cas9 technology increased the resistance of this strain to compound **11**. Mass spectrometry-based proteomics (LC–MS/MS) of wild-type tubercle bacilli growing in subinhibitory concentrations of compounds **11** or **15** revealed 15 overproduced proteins not detectable in the control cells, including virulence-related proteins.

## Introduction

*Mycobacterium tuberculosis*, the causative agent of tuberculosis (TB), is one of the most serious bacterial pathogens, claiming 1.5 million lives each year. Tubercle bacilli are intracellular pathogens, and their life cycle includes long states of persistence. Therefore, *M. tuberculosis* is relatively hard to eradicate and poses a challenge for effective chemotherapy. Tuberculosis therapy lasts 6–24 months, depending on the drug susceptibility of the infecting bacterial strain and its metabolic state. In particular, the treatment of multidrug-resistant tuberculosis (MDR-TB) is very expensive, very difficult for patients to follow, carries a burden of side effects and is successful in only approximately 54% of cases (Jagielski et al., 2016). However, even tuberculosis caused by drug-sensitive strains requires 6 months and a cocktail of four drugs used daily to prevent the selection of drug-resistant *M. tuberculosis* mutants. The rise of drug resistance among *M. tuberculosis* strains in recent years and the phenomenon of HIV (human immunodeficiency virus)*–M. tuberculosis* coinfection are serious public health challenges worldwide. Therefore, the development of alternative medical strategies based on a new generation of drugs is desperately needed to effectively cure MDR-TB, reduce the duration of current therapies and minimize the toxicity and cost of the antituberculosis agents used (Zumla et al., 2015). A new hope for the improved treatment of MDR-TB is based on bedaquiline and delamanid, drugs recently approved by American and European drug agencies (FDA/EMA) that are under investigation in clinical trials (https://clinicaltrials.gov/ct2/show/NCT02754765). Delamanid is a promising nitroimidazole with the potential to decrease the duration of anti-TB treatment, thereby reducing the mortality rate of MDR-TB (Ryan and Lo, 2014). Bedaquiline is a diarylquinolone with activity against MDR-TB that is able to kill both actively replicating and dormant mycobacteria, thus shortening the duration of a treatment regime (Koul et al., 2008).

An interesting group of compounds with wide biological activity, including antibacterial, antiviral, cytotoxic, anticonvulsant, and analgesic activities, are thiosemicarbazides (Nevagi et al., 2014; Cihan-Ustundag et al., 2016). These compounds carry sulfur and nitrogen atoms and can easily form hydrogen bonds with proteins. Some derivatives of thiosemicarbazides, such as 2-butyl-4-chloroimidazole-based substituted piperazine-thiosemicarbazone hybrids, manganese complexes derived from 2-acetylpyridine-N (4)-R-thiosemicarbazones and 4-nitropyrrole-semicarbazide conjugates, have been reported due to their anti-*M. tuberculosis* activity (Jallapally et al., 2014; Oliveira et al., 2014; Rane et al., 2014). More recently, three of 30 investigated imidazole-thiosemicarbazide derivatives appeared to be active against *M. tuberculosis in vitro*, with only one found to be active against bacilli that had been engulfed by human macrophages (Bekier et al., 2021). The selected compounds also presented inhibitory activity against mycobacterial biofilms.

The potent FtsZ inhibitor (E20) carrying a 2,4-disubstituted-6- thiophenyl-pyrimidine and a chiral amino quinuclidine moiety was not effective against bacteria in the phenotypic screening (Chan et al., 2013). On the other hand, 2,4-disubstituted-6-thiophenyl-pyrimidine derivative (F20) showed antibacterial activity against Gram-positive bacteria, including methicillin-resistant *Staphylococcus aureus* (MRSA) and vancomycin-resistant *Enterococcus* faecium (VREF). It was reported that F20 inhibits the activity of GTPase, disrupts FtsZ polymerization, and finally impairs bacterial cell division leading to cell death (Fang et al., 2019). Other compounds carrying pyrimidine ring linked to the benzimidazole orbenzothiazole moieties through an acrylonitrile bridge, were evaluated for their *in vitro* antibacterial activity against *S. aureus*, *Bacillus* subtilis, *Escherichia coli*, and *Pseudomonas aeruginosa*. The docking studies indicated that the compounds inhibit the β-lactamase enzyme through covalent bonding with Ser70 (AlNeyadi et al., 2017). A new class of azolyl pyrimidines linked by diamino sulfone moiety displayed antimicrobial (*B. subtilis*) and antifungal (Aspergillus niger) activity (Butta et al., 2016). The high-throughput screening allowed to select 190 substituted simple or fused pyrimidines with modest activity against bacterial growth, including *M. tuberculosis*, but many active compounds showed significant cytotoxicity. Substituted pyrimidines 2-(3,5-dimethyl-pyrazol-1-yl) group with various 4-phenylamino and 4-cycloakylamino and 5-carboxyethyl substituents showed higher activity/selectivity ratios than closely related pyrimidines substituted with 2-(2-pyridyl), 4- phenylthio, and 5-methoxy groups (Maddry et al., 2009).

Nineteen novel cycloalkyloaminothiosemicarbazide derivatives were synthesized recently and evaluated against *M. tuberculosis*. Compounds carrying a morpholine ring at the end of the thiosemicarbazide chain exhibited activity against tubercle bacilli growing *in vitro*. One compound presented high specificity against *M. tuberculosis* and very low cytotoxicity, as determined for human dermal fibroblasts and mouse melanoma cell lines (Krause et al., 2020).

Here, we selected two of the most promising cycloalkyloaminothiosemicarbazide derivatives to test their activity against tubercle bacilli localized inside human macrophages, as well as against biofilm-forming *M. tuberculosis.* The frequency of mutations resulting drug-resistant strains was also determined. Whole-genome sequencing analysis of resistant mutants allowed us to identify that mutations accumulated in the *mmpR5* gene increased resistance to the tested compounds, as well as to the EMA/FDA approved drug bedaquiline.

## Materials and Methods

### Compounds

The presented compounds, from a chemical point of view, belong to the group of 2,4-disubstituted pyridine derivatives (Krause et al., 2020). In the 2-position, they have a thiosemicarbazone group linked to a hydrazonamide moiety. Compounds were analyzed using the SwissADME service (Swiss Institute of Bioinformatics 2021) (Daina and Zoete, 2016; Daina et al., 2017) and ProTOX II service (Banerjee et al., 2018) to obtain computational pharmacokinetic and toxicological profiles. Both of the compounds are predicted to have a high absorption in the gastrointestinal tract, which may make them effective drugs (Table 1, Supplementary Figure S1).

**TABLE 1:** Compounds used in this work.

Compounds	Bioavailability radar	Predicted LD50 [mg/kg] (predicted toxicity class)
**11, LogP 2.56** 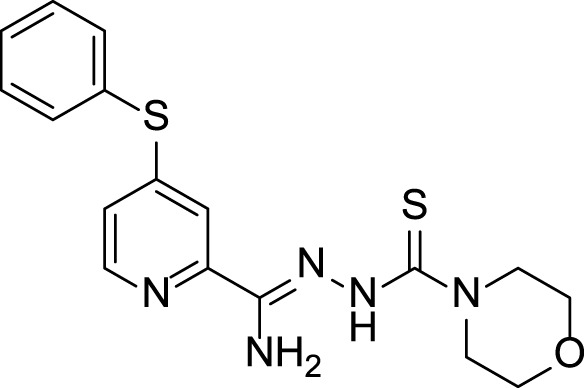	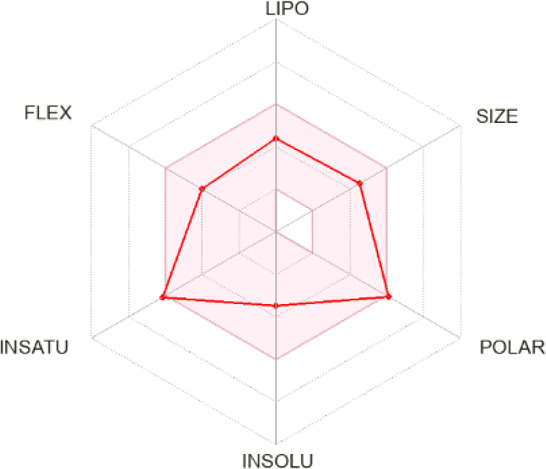	70 (3) for neutral form
		3500 (5) for zwitterion
**15, LogP 1.06** 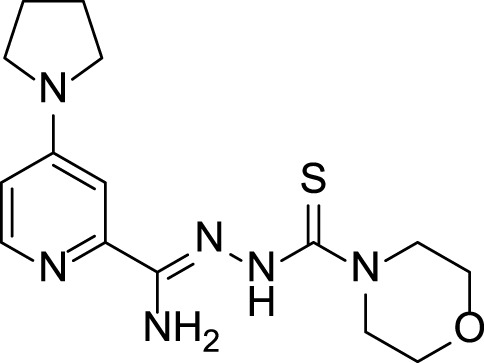	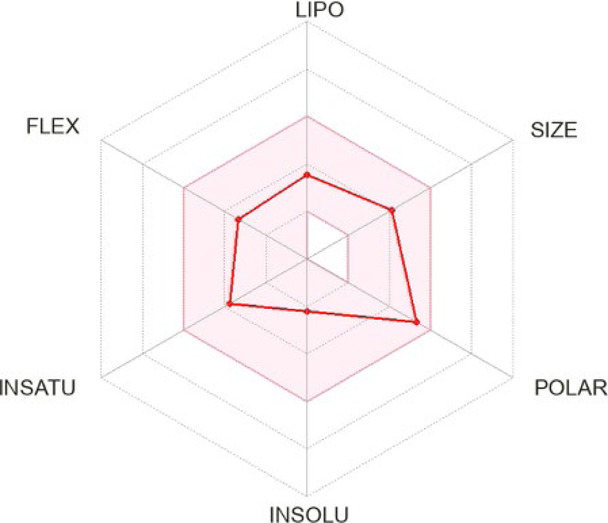	3 (1) for neutral form
		1,600 (4) for zwitterion

Bioavailability radar was used to determine the drug-likeness of the tested compounds. The prediction LD50 method is based on the analysis of the two-dimensional similarity to compounds with known LD50 values and the identification of fragments overrepresented in toxic compounds. LD50 values are given in [mg/kg]. Class I: fatal if swallowed (LD50 ≤ 5), Class II: fatal if swallowed (5 < LD50 ≤ 50), Class III: toxic if swallowed (50 < LD50 ≤ 300), Class IV: harmful if swallowed (300 < LD50 ≤ 2000), Class V: may be harmful if swallowed (2000 < LD50 ≤ 5,000), Class VI: nontoxic (LD50 > 5,000).- lipophilicity (LIPO) is within the range -0.7 < XlogP3 < +5.0; molecular weight (SIZE) is 150 g/mol < MW < 500 g/mol; polarity (POLAR) is 20 Å^2^ < TPSA <130 Å^2^; insolubility (INSOLU) is 0 < logS <6; insaturation (INSATU) is 0.25 < fraction Csp3 < 1; and flexibility (FLEX) is 0 < Num rotatable bonds <9). For drug-like properties, compounds were found to have a good bioavailability score (0.55) (Lipinski et al., 2012)^,^ which is consistent with Lipiński’s rule of five. This compound meets the rules of Lipinski, Ghose, Veber and Muegge (Ghose et al., 1999; Muegge et al., 2001; Veber et al., 2002; Lipinski et al., 2012). Shown in the BOILED-Egg diagram, the test samples do not pass the blood–brain barrier but are absorbed in the gastrointestinal tract. The compounds are not available through the skin, as indicated by a negative logKp value (-7.84 cm/s). The ProTox II, webserver classified the toxicity classes of the ligands.

### Bacterial strains and growth conditions

*M. tuberculosis* H_37_Rv was grown at 37°C in Middlebrook 7H10 (Difco, Baltimore, MD, United States) medium supplemented with 10% OADC (oleic acid-albumin-dextrose-catalase). The liquid cultures were grown in Middlebrook 7H9 broth (Difco, Baltimore, MD, United States) supplemented with OADC and 0.05% Tween-80 (pH = 7).

The MIC_90/50_ for *M. tuberculosis* was determined on solid and/or liquid media supplemented with various concentrations of the tested chemical agents. The 2,4-disubstituted pyridine derivatives were dissolved in dimethyl sulfoxide (DMSO) and added directly to the growth medium. The final concentration of DMSO in the medium never exceeded 0.1% (vol/vol), and DMSO did not affect the growth of the bacilli.

The Microplate Alamar Blue Assay (MABA test) was applied to define the MIC value as described by Franzblau et al. (1998). The susceptibility of the tested strains was assessed based on the change in color from blue to pink based on visual inspection. Wells containing only bacteria, medium, or compound were used as controls in this experiment, and the MABA test was repeated independently three times.

### Selection of mutants and determination of mutation frequency

The mutants resistant to the tested compounds were selected on 7H10/OADC solid media supplemented with the **11** (6 and 8 μg/ml) and **15** (9 and 15 μg/ml) compounds. The frequency of resistance to **11** and **15** was determined as described previously (Korycka-Machala et al., 2019). Briefly, approximately 300,000 cells of *M. tuberculosis* culture at an OD of 0.8–1.0, were used to inoculate 100 ml of Middlebrook 7H9 supplemented with 10% Middlebrook OADC, 0.05% tween 80, and 0.005% glycerol, giving a total cell count of 10,000 cells per 4 ml culture. This volume was divided to start 20 cultures of 4 ml each. Cultures were grown at 37°C until reaching an OD of 1.0. Then the cultures were spun at 4000 RPM for 10 min at 4°C and resuspended in 250 μl of 7H9/OADC/tween/glycerol and spotted onto 7H10/OADC/tween/glycerol plates supplemented with 0.5, 2.0 μg/ml rifampicin, 2.0 μg/ml streptomycin, 6.0, 8.0 μg/ml **11,** and 9.0, 15.0 μg/ml **15**.

### Biofilm formation assay

The effect of compounds **11** and **15** on mature mycobacterial biofilms was determined as described previously (Bekier et al., 2021). Briefly, to develop the biofilm, *M. tuberculosis* was cultured to an OD_600_ = 1 in 7H9/OADC supplemented with 0.05% Tween 80. Next, the inoculum was added at a ratio of 1:100 v/v to Sauton’s medium, and then the obtained mixture was dispensed into each well of a 24-well plate (2.5 ml/well). The plate was covered with a lid, protected with parafilm, and incubated in humidity at 37°C for 5 weeks. Next, the medium under the mature biofilm was replaced with 2.5 ml of Sauton’s medium supplemented with 0.1% casitone and the compounds in various concentrations (compounds were not added to controls). The plates were then covered, sealed with parafilm, and incubated at 37°C for 48 h. The viability of the bacilli was determined by fluorescence measurements (excitation: 550 nm, emission: 590 nm) in the presence of resazurin (375 µl of 0.02% resazurin per well) after 90 min of incubation using the multimode microplate reader SpectraMax^®^ i3 (Syngen). The no compound control was used to represent 100% viability. The results were expressed as the percent viability compared to untreated *Mycobacterium* biofilm. We also investigated the effect of compounds **11** and **15** on biofilm formation. In the analysis, the diluted inoculum of *M. tuberculosis* in Sauton’s medium was supplemented with 0.6 and 0.4 μg/ml of compounds **15** and **11**, respectively, and dispersed into each well (2.5 ml). The plates were protected by parafilm and incubated in a humidified incubator at 37°C for 5 weeks. The biofilm was photographed and the medium was replaced with 2.5 ml of Sauton’s medium supplemented with 0.1% casein hydrolysate and 375 µl of a 0.02% resazurin solution, and the plate was incubated for 90 min. Further, 200 µl of each sample was transferred to a 96-well plate, and the amount of resorufin was detected using a SpectraMax^®^ i3 multimode microplate reader (Molecular Devices, San Jose, CA, United States) at excitation 560 nm, emission 590 nm. No compound samples were used as a control of 100% viability.

### Preparation of human MDMs, *in vitro* cytotoxicity assay, and evaluation of the bactericidal effect of the compounds 11 and 15 on intracellularly growing tubercle bacilli

Human monocytes were isolated from commercially available (Regional Blood Donation Station, Lodz, Poland) and freshly prepared buffy coats from healthy human blood donors (Korycka-Machala et al., 2019; Kawka et al., 2021). The cultures of differentiated human monocyte-derived macrophages (MDMs) were extensively washed to remove any nonadherent cells, left resting overnight, and incubated with culture medium supplemented with various concentrations of the tested compounds. The viability of macrophages was determined after 48 h of incubation with 3-(4,5-dimethylthiazol-2-yl)-2,5-diphenyltetrazolium bromide (MTT) (Sigma, St. Louis, MO, United States), as described previously (Korycka-Machala et al., 2017). Additionally, the MDMs were infected with tubercle bacilli at an MOI of 1:10 as described by Korycka-Machala et al. (2017). Two hours after infection, the extracellularly located bacteria were extensively washed out with culture medium, followed by an additional 1 h incubation in medium supplemented with 1 g/L gentamicin (Sigma, St. Louis, MO, United States); finally, the samples were washed three times with Iscove’s medium supplemented with 2% human AB serum (Sigma, St. Louis, MO, United States). Next, culture medium with or without (control) the test compounds at a concentration of 1x MIC_90_ was added to independent cultures of the infected macrophages, followed by incubation at 37°C for 48 h under a humidified atmosphere of 10% CO_2_–90% air. Finally, the macrophages were lysed with 0.1% SDS, and the number of CFUs (colony forming units) was determined as previously described (Korycka-Machala et al., 2020).

### Whole-genome sequencing

The sequencing libraries were prepared using the Nextera XT DNA sample preparation protocol (Illumina Inc., San Diego, CA, United States). A total of 1 ng of genomic DNA isolated from the wild-type and ten individual mutants was used for the preparation of paired-end libraries according to the manufacturer’s instructions. Whole-genome shotgun sequencing and *in silico* analysis were performed as described previously (Korycka-Machala et al., 2019; Korycka-Machala et al., 2020). Sequences of the seven mutants selected for compound **11** and the three mutants selected for compound **15** have been deposited in GenBank under accession no. PRJNA843930.

### CRISPR/Cas9

The depletion of the MmpR5 protein in *M. tuberculosis* was performed by applying the CRISPR/Cas9 strategy optimized by Rock et al. (2017). The sgRNA probe carrying 20 nucleotide target sequences followed by a strong (GGAAA) PAM site was introduced into the chromosomal DNA using the pLJR965 integration vector (Supplementary Figure S2). The silencing of *mmpR5* transcripts in the presence of the inducer anhydrotetracycline (aTc, 100 ng/ml) was confirmed by qRT–PCR. RNA was isolated from induced and control cultures using TRIzol LS reagent (Invitrogen, Carlsbad, CA, United States) and mechanical disruption (FastPrep-24, MP Biomedicals Eschwege, Germany). qRT–PCR analysis was performed as described previously using *sigA* gene expression as the internal control (Korycka-Machala et al., 2020). The relative fold change was determined using the double delta Ct method (2^−ΔΔCT^). The reaction was carried out at 60°C. The 208 bp in length *sigA* product was obtained using RvsigA-F (CCTACGCTACGTGGYGGATTCG) forward and RvsigA-R (TGG​ATT​TCC​AGC​ACC​TTC​TCC​G) reverse primers. The 136 bp in length *mmpR5* product was obtained using RvmmpR5-F (TGG​CTG​ACG​TGG​GGC​TGA​GG) forward and RvmmpR5-R (CCG​GTT​CGC​TGG​CTG​TAT​CGC) reverse primers, the 78 bp in length *mmpS5* was obtained using RvmmpS5-F (ATC​ACC​TGC​CGA​ATC​ACC​GT) forward and RvmmpS5-R (GCA​GTA​GGT​CAG​GGC​ATC​CA) reverse, the 181 bp in length *mmpL5* using RvmmpL5-F (ACA​CCC​TCG​ACG​GAA​TCG​AC) forward and RvmmpL5-R (TGA​TCC​TGC​AGC​CCT​TCC​TG) reverse primers.

### Proteomics data analysis

The mass spectrometry analyses of the whole-cell protein lysates obtained according to previously published methodologies (Plocinski et al., 2019) were performed as a service at the Institute of Biochemistry and Biophysics PAS on a Q Exactive high-performance mass spectrometer using an experimental pipeline reported elsewhere (Goralczyk-Binkowska et al., 2020). The mass spectrometry proteomics data have been deposited to the ProteomeXchange Consortium *via* the PRIDE (Perez-Riverol et al., 2022) partner repository with the dataset identifier PXD035807.

## Results and discussion

### 2,4-Disubstituted pyridine derivatives are effective against intracellularly located *M. tuberculosis*

4-Substituted picolinohydrazoamides were reported as a new class of antitubercular agents active against tubercle bacilli at low concentrations and presenting limited cytotoxicity on human dermal fibroblasts and mouse melanoma cell lines (Krause et al., 2020). The two compounds, **11** and **15**, differ only in the substituents at the 4-position of the pyridine ring. However, the nature of these substituents greatly influences the LogP values associated with bioavailability and the ease of penetration through biological barriers. The pyrrolidine-containing derivative **15** is less lipophilic (LogP 1.06). The nitrogen atom present in the pyrrolidine group shows basic properties, which translates into the ability to bind protons and thus better interaction with proton donating targets. The LogP value for derivative **11** (2.56) is within the range of 1.3–4.1 required for potential anti-tuberculosis chemotherapeutic agents (Santos et al., 2020), but the sulfur atom present in it does not show a tendency to be protonated. Hence, presumably, there is a difference in the antimycobacterial activity observed for the two compounds (Table 1).

**FIGURE 1:**
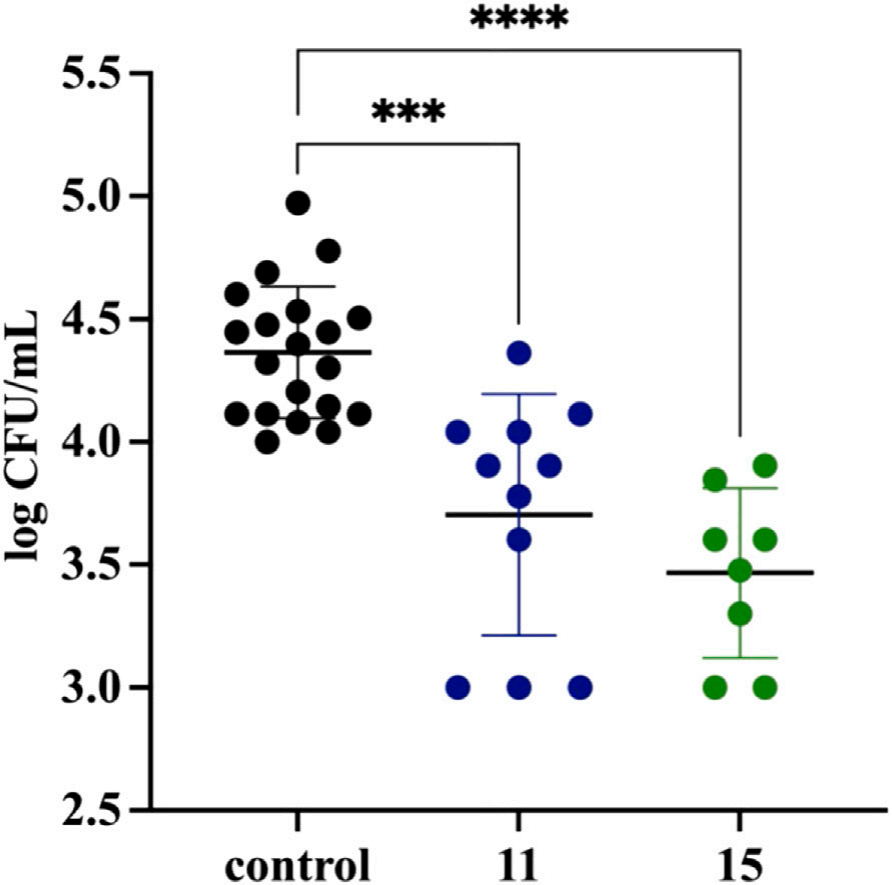
Inhibitory effect of compounds 11 and 15 on the intracellular growth of M. tuberculosis in human monocyte-derived macrophages. The intracellular growth of the pathogen in the experimental (11/15-treated) samples versus the control (untreated) sample is presented as a log CFU/mL, which represents the ratio of the CFU number determined for the 11 (blue) and 15 (green) treated samples to the CFU number estimated in the control sample. Error bars indicate standard deviations. Data were analyzed using the Mann–Whitney U test. The differences between control cells and compound-treated samples were statistically significant, with p = 0.0006 (***) and *p* < 0.0001 (****) for compounds 11 and 15, respectively. The statistical analysis was done using SigmaStat v. 4.0.

*Mycobacterium tuberculosis* is an intracellular pathogen able to propagate inside host alveolar macrophages during infection, where it is protected from drugs unable to penetrate inside human phagocytes. Here, we evaluated the activity of the two most potent compounds, **11** and **15**, against intracellularly located tubercle bacilli. The optical density (OD_600_) of *M. tuberculosis* in *vitro* culture and CFU analyses confirmed the strong *in vitro* antitubercular effect of both compounds, with MIC_99_ values as low as 0.8 and 1.5 μg/ml for **11** and **15**, respectively (Supplementary Figure S3). MDMs were differentiated from peripheral blood monocytes that were isolated from buffy coats of healthy human blood donors and used to determine the cytotoxicity of the investigated compounds. Compounds **11** and **15** exhibited cytotoxic effects (IC_50 values_) against human phagocytes at concentrations that exceeded 1.2 and 1.5 μg/ml, respectively (Supplementary Table S1). To test the activity of the investigated compounds against *M. tuberculosis* engulfed by human macrophages, tubercle bacilli at an MOI of 1:10 were used for infection of the MDMs. After 2 h of phagocytosis, the extracellular bacteria were washed out, and the remaining cell membrane-attached bacilli were killed by gentamicin. The macrophages carrying *M. tuberculosis* were then exposed to the compounds at a 1x MIC concentration and incubated for 48 h. The number of viable, intracellularly located bacilli was determined by CFU enumeration. A significant (*p* ≤ 0.00064, *p* ≤ 0.0001) decrease in the number of viable bacilli was observed in the macrophages treated with compounds **11** and **15**, respectively (Figure 1), compared to the control cells, which indirectly suggests an ability of the compounds to cross the target cell membrane. Efficient penetration inside macrophages was also previously reported for compounds such as thio-functionalized carbohydrate derivatives (Korycka-Machala et al., 2017), 1H-benzo [d]imidazole derivatives (Korycka-Machala et al., 2019), and imidazole-thiosemicarbazide derivatives (Bekier et al., 2021).

### The *M. tuberculosis* biofilm is sensitive to 2,4-disubstituted pyridine derivatives

**FIGURE 2:**
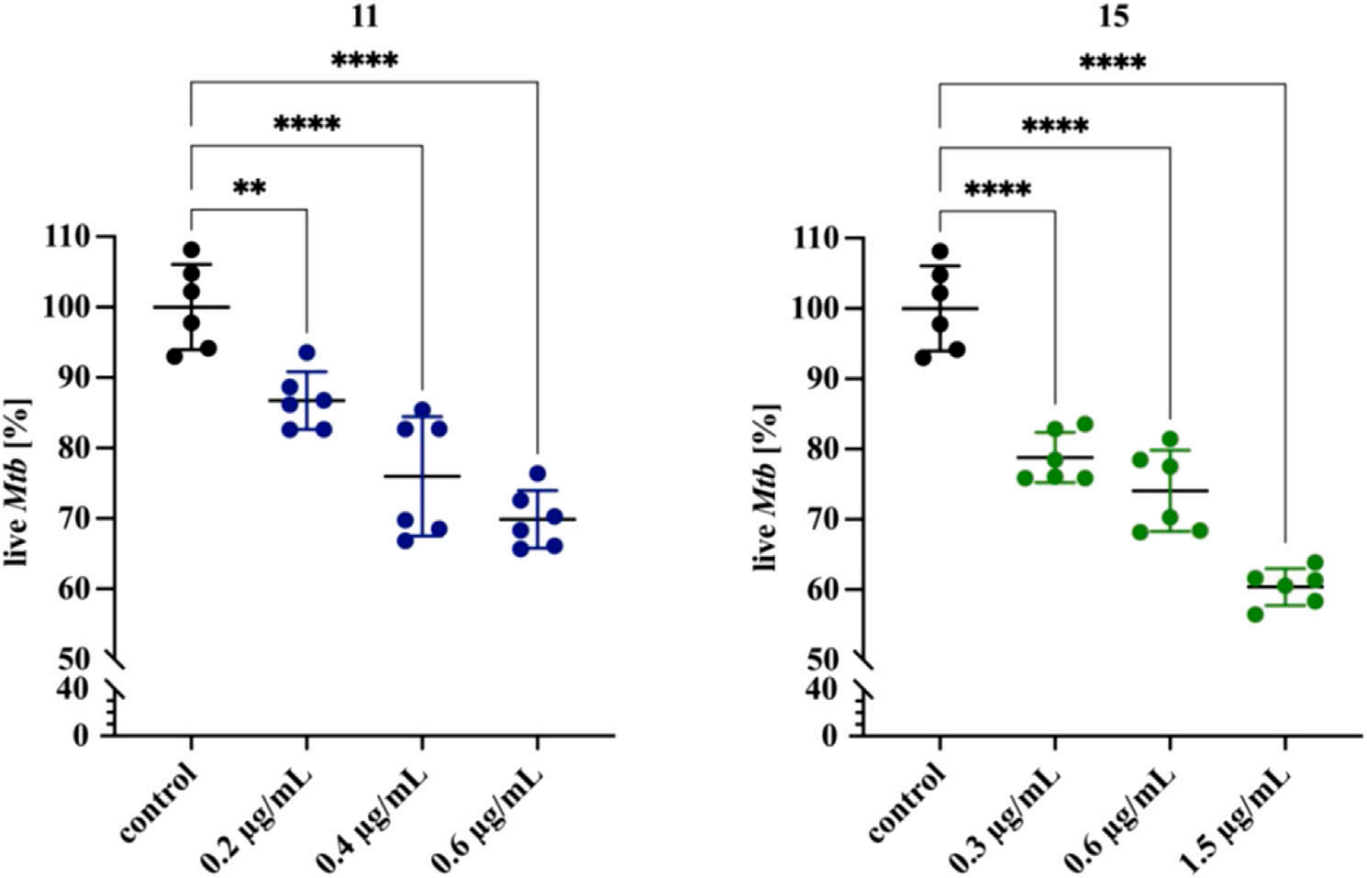
Effects of compounds 11 and 15 on mature biofilms composed of M. tuberculosis. Error bars indicate the standard error of the mean (SD). ** depicts the value with significant differences at p=0.0027, **** depicts the values with significant differences at p<0.0001. Data were compared using one-way ANOVA followed by Dunnett’s test. No compound control is represented by black dots. Blue and green dots represent compound 11 and 15, respectively. The concentrations of compounds are depicted at X-axis. The statistical analysis was done using SigmaStat v. 4.0. The graph was prepared using GraphPad Prism 9 version 9.3.1 (350).

The specific composition of the cell wall allows mycobacteria to adhere to surfaces, as well as to form biofilms in the air–media interfaces (Esteban and Garcia-Coca, 2017; Dokic et al., 2021), making them prevalent in almost all environments. Antibiotics have difficulty penetrating biofilms, mainly because the bacteria in the biofilm are in a dormant form, but also because the biofilm creates anaerobic areas where some compounds are not active; these factors favor the development of drug resistance. The drug resistance of biofilm-forming microorganisms, including *M. tuberculosis,* may result in treatment failure with drugs that are normally active against the same bacteria in the planktonic state (Islam et al., 2012). It was reported that *M. tuberculosis* can develop a biofilm *in vitro* (Ojha et al., 2008); however, its role in the pathogenesis of *tuberculosis* has not yet been elucidated. Biofilms composed of tubercle bacilli *in vivo* are considered to be due to caseous necrosis and cavitation formation in the lungs. Therefore, potential new anti-TB drugs should present bactericidal effects against intra- or extracellularly located planktonic bacilli, as well as against biofilm-forming bacteria. To test whether compounds **11** and **15** are active against *M. tuberculosis* in a biofilm, we grew bacteria for 5 weeks in Sauthon’s medium as described in the Methods section. Next, the medium under the biofilm was replaced with a fresh medium supplemented or not with the investigated compounds, and the cultures were further incubated for 48 h. The resazurin-based determination of the viability of the tubercle bacilli in the tested and control biofilm cultures allowed us to identify the dose-dependent bactericidal effect of both compounds on the biofilm composed of *M. tuberculosis*. The presence of 0.2, 0.4, and 0.6 μg/ml of compound **11** decreased the viability of *M. tuberculosis* biofilms by approximately 13, 24, and 30%, respectively (*p* < 0.0027, *p* < 0.0001 for 0.2 and 0.4–0.6, respectively). Compound **15** affected the viability of biofilm-forming bacilli at concentrations of 0.3, 0.6, and 1.5 μg/ml by approximately 21, 26, and 40%, respectively (*p* < 0.0001) (Figure 2, Supplementary Table S2). Further, we investigated the effect of compounds 11 and 15 on biofilm development. The supplementation of media with 0.4 and 0.6 μg/ml of compounds **11** and **15**, respectively inhibited the biofilm formation. The effect was more pronounced for compound **11** with about 80% of decrease in the viability of tubercle bacilli (Supplementary Figure S4). A similar analysis was made by Bekier and others for the imidazole-thiosemicarbazide derivative (ITD-13). The investigated compound at the MIC_90_ concentration was able to inhibit the formation of biofilms and decrease the tubercle bacilli viability by approximately 90%. However, mature biofilm treated with ITD-13 at a 1x MIC concentration decreased the viability of the bacilli by only approximately 15% (Bekier et al., 2021).

### Mutants resistant to 2,4-disubstituted pyridine derivatives are not viable in broth culture

The acquired resistance of *M. tuberculosis* to anti-TB drugs is typically due to the accumulation of mutations in a gene encoding a drug target, an enzyme activating a given prodrug within the bacilli, or a protein involved in the transport of a drug. The frequency of acquiring resistance to particular anti-TB drugs differs depending on the number of possible mutations that can result in drug resistance (David, 1970). The calculated mutation rate for both investigated compounds was similar to that identified for the most potent anti-TB drug rifampicin and much higher compared to streptomycin (Table 2). Moreover, the mutants selected on plates supplemented with compounds **11** or **15** at a concentration of 10x MIC were not viable when inoculated in broth carrying the same concentrations of compounds.

**TABLE 2:** The mutation rate of tubercle bacilli calculated for the investigated compounds and anti-TB drugs.

Compound/concentration (µg/ml)	Mutation rate ±SD
**11/**6	2.7 × 10^–9^ ± 1.75 × 10^–9^
**11/**8	1.3 × 10^–9^ ± 3.0 × 10^–8^
**15/**9	5.5 × 10^–9^ ± 2.0 × 10^–9^
**15/**15	7.0 × 10^–10^ ± 3.4 × 10^–10^
Rifampicin/0.5	5.5 × 10^–8^ ± 1.7 × 10^–8^
Rifampicin/2.0	1.8 × 10^–9^ ± 7.1 × 10^–7^
Streptomycin/2.0	3.5 × 10^–7^ ± 3.0 × 10^–7^

The Next Generation Sequencing (NGS) of whole DNA isolated from seven mutants grown on 7H10/OADC supplemented with 8 μg/ml of compound **11** and 3 mutants grown in the presence of 9 μg/ml of compound **15** revealed that all investigated mutants resistant to **11** carried mutations in various locations in the *rv0678* gene coding for the MmpR5 protein (Table 3 and Supplementary Table S3). One of three mutants selected in the presence of compound **15** carried a nucleotide substitution in the gene encoding the MmpL5 protein, which is controlled by the MmpR5 regulator and was reported to be part of the MmpS5-MmpL5 efflux system related to azole resistance in *M. tuberculosis* (Milano et al., 2009). None of the selected mutants carried mutations in an essential gene or in a gene encoding an enzyme that could modify the compounds used. The very low frequency of acquired resistance to compounds **11** and **15**, the viability of the mutants exclusively on solid media, and the accumulation of mutations in the putative efflux-pump system suggested that the resistance phenotype was due to the enhanced detoxification of the bacilli carrying mutations in either the *mmpR5* or *mmpL5* genes*.* The lack of mutations in genes encoding a target protein(s) could suggest a multitarget mode of action for both compounds used. In such a case, the selection of resistant mutants would require mutations of more than a single target and might happen with lower efficiency than a mutation enhancing the detoxification of bacilli by the efflux-pump system.

**TABLE 3:** The mutations identified in *mmpR5* (*rv0678*) and *mmpL5* (*rv0676c*) genes of mutants resistant to compounds **11** and **15**.

Compound/Number	Position	Mutation	Gene
11/1	779.187	(G)6→5	Rv0678
11/2	779.389	+C	Rv0678
11/3	779.389	+C	Rv0678
11/4	779.273	T→C	Rv0678
11/5	779.294	Δ1 bp	Rv0678
11/6	779.326	G→A	Rv0678
11/7	779.326	G→A	Rv0678
15/1	776.165	G→T	Rv0676c

**FIGURE 3:**
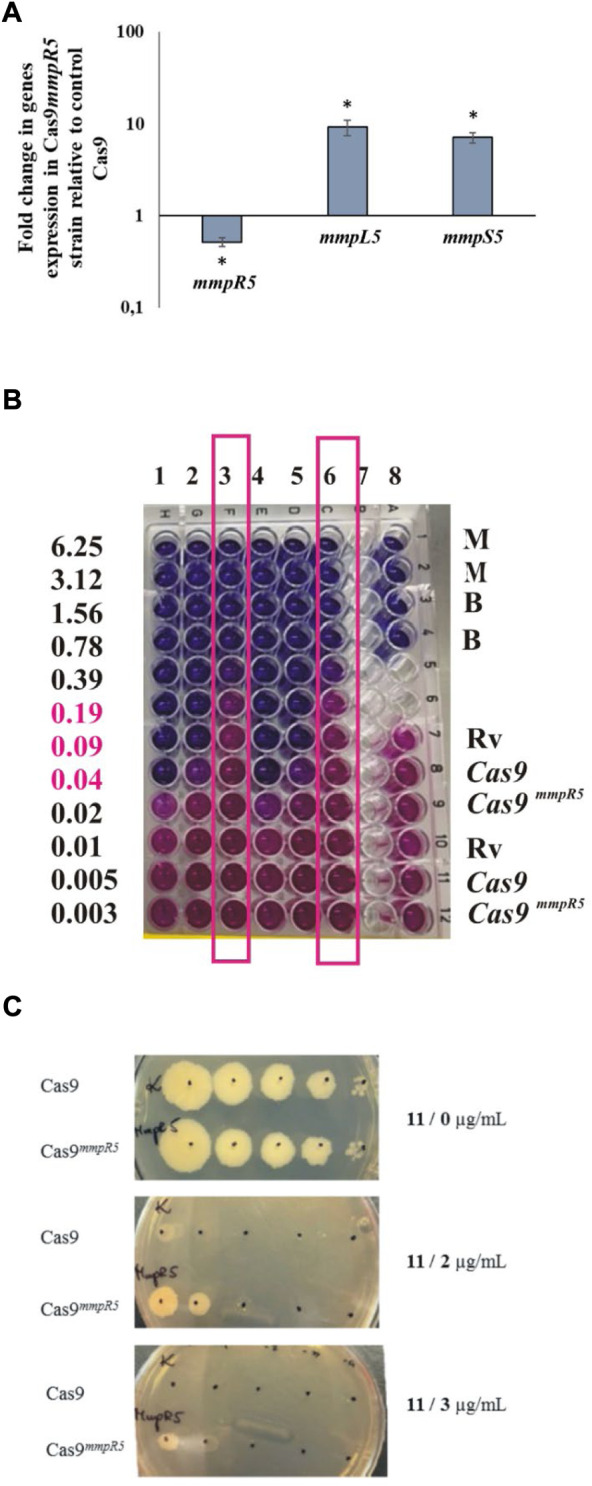
Depletion of MmpR5 affects the resistance of M. tuberculosis to compounds 11, 15, and bedaquiline. **(A)** Expression profile of mmpR5, mmpL5, mmpS5 genes in CRISPR Cas9mmpR5 M. tuberculosis mutant. The mutant M. tuberculosis strain CRISPR–Cas9mmpR5 (Cas9 mmpR5) and the control CRISPR–Cas9 strain expressing the “empty” CRISPRi/dCas9 vector (cas9) were grown in 7H9/OADC broth for 16 h at 37°C with the addition of anhydrotetracycline (100 ng/μl) to deplete MmpR5. Transcript levels were determined using qRT–PCR and SYBR green chemistry. The expression level of mmpR5, mmpL5, mmpS5 was normalized to the sigA housekeeping gene and compared to the control strain. The calibrator was the control strain carrying an empty vector, + aTc. Statistical significance was determined using Student’s t test (mmpR5 -p = 0.007663, mmpS5 -p = 0.0004, mmpL5 -p =0.0014 ) and Microsoft Excel/Office 365. **(B)** The depletion of MmpR5 affects the resistance of tubercle bacilli to bedaquiline (BDQ) as determined by the Alamar blue assay. Columns 1 and 4, M. tuberculosis H37Rv + BDQ; columns 2 and 5, M. tuberculosis CRISPR–Cas9 + BDQ; columns 3 and 6, M. tuberculosis CRISPR–Cas9mmpR5 + BDQ; column 8, controls: M (7H9 medium), B (BDQ µg/mL), Rv (M. tuberculosis H37Rv), Cas9 (M. tuberculosis CRISPR–Cas9), Cas9mmpR5 (M. tuberculosis CRISPR–Cas9mmpR5). Columns 3 and 6 representing M. tuberculosis CRISPR–Cas9mmpR5 + BDQ were framed. The growth of bacteria (pink color) detected at concentrations of BDQ 0.19, 0.09, and 0.04 (pink fonts) was detected for M. tuberculosis CRISPR–Cas9mmpR5 + BDQ exclusively. **(C)** Growth on solid medium in the presence of compound 11. Growth of M. tuberculosis CRISPR–Cas9 (Cas9) and M. tuberculosis CRISPR–Cas9mmpR5 (Cas9mmpR5) on the control 7H10/OADC plate (1) and on plates supplemented with 2 µg/mL (2) and 3 µg/mL (3) of compound 11.

The inactivation of MmpR5, which leads to the upregulation of the MmpS5-MmpL5 efflux pump, was related to low level resistance to various drugs, including clofazimine and bedaquiline (Hartkoorn et al., 2014). Therefore we decided to use the bedaquiline to confirm the phenotype of CRISPR–Cas9^*mmpR5*^ mutant. MmpS5-MmpL5 efflux system affects particular compounds acting on or affected by the electron transport chain (azoles, clofazimine, and bedaquiline). However, it was reported (Villellas et al., 2017) that MmpS5-MmpL5 mutations affect the resistance of *Mtb* also to telithromycin and partially to rifampicin (not statistically significant) but not to rifabutin, amikacin, ofloxacin, streptomycin, and isoniazid. We verified the role of the mutations in *mmpR5* in the acquired resistance to the investigated compounds by constructing an *M. tuberculosis* CRISPR–Cas9 mutant with an inducible (anhydrotetracycline, aTc) depletion of the MmpR5 regulator. *M. tuberculosis* mutants carrying a 20-nucleotide *mmpR5* target sequence followed by a strong PAM site were analyzed by qRT–PCR to determine the *mmpR5, mmpL5* and *mmpS5* mRNA levels in the presence and absence of the inducer (aTc). The supplementation of the *M. tuberculosis* CRISPR–Cas9^*mmpR5*^ culture with aTc depleted the mRNA for *mmpR5* by approximately 50%. Moreover, we observed the 10x increased expression for transcripts of *mmpL5* and *mmpS5* in *M. tuberculosis* CRISPR–Cas9^*mmpR5*^ strain (Figure 3A). On the other hand, the control strain carrying an empty CRISPR–Cas9 vector was not affected in the presence of aTc. By applying the Alamar-Blue assay, we also observed an increased resistance of *M. tuberculosis* CRISPR–Cas9^*mmpR5*^ to bedaquiline in the presence of aTc, as expected. The MIC, determined to be 0.04 μg/ml for the wild-type strain, increased to 0.39 μg/ml for the CRISPR–Cas9^*mmpR5*^ mutant (Figure 3B). Furthermore, we used the CRISPR–Cas9^*mmpR5*^ mutant to determine the MIC value for compound **11** in the MmpR5 depletion background. The MIC was determined on 7H10/OADC plates supplemented with aTc and the investigated compound and was found to increase from 0.4 to 3 μg/ml in the presence of the inducer (Figure 3C).

### Proteomic response to subinhibitory concentrations of 2,4-disubstituted pyridine derivatives

**FIGURE 4:**
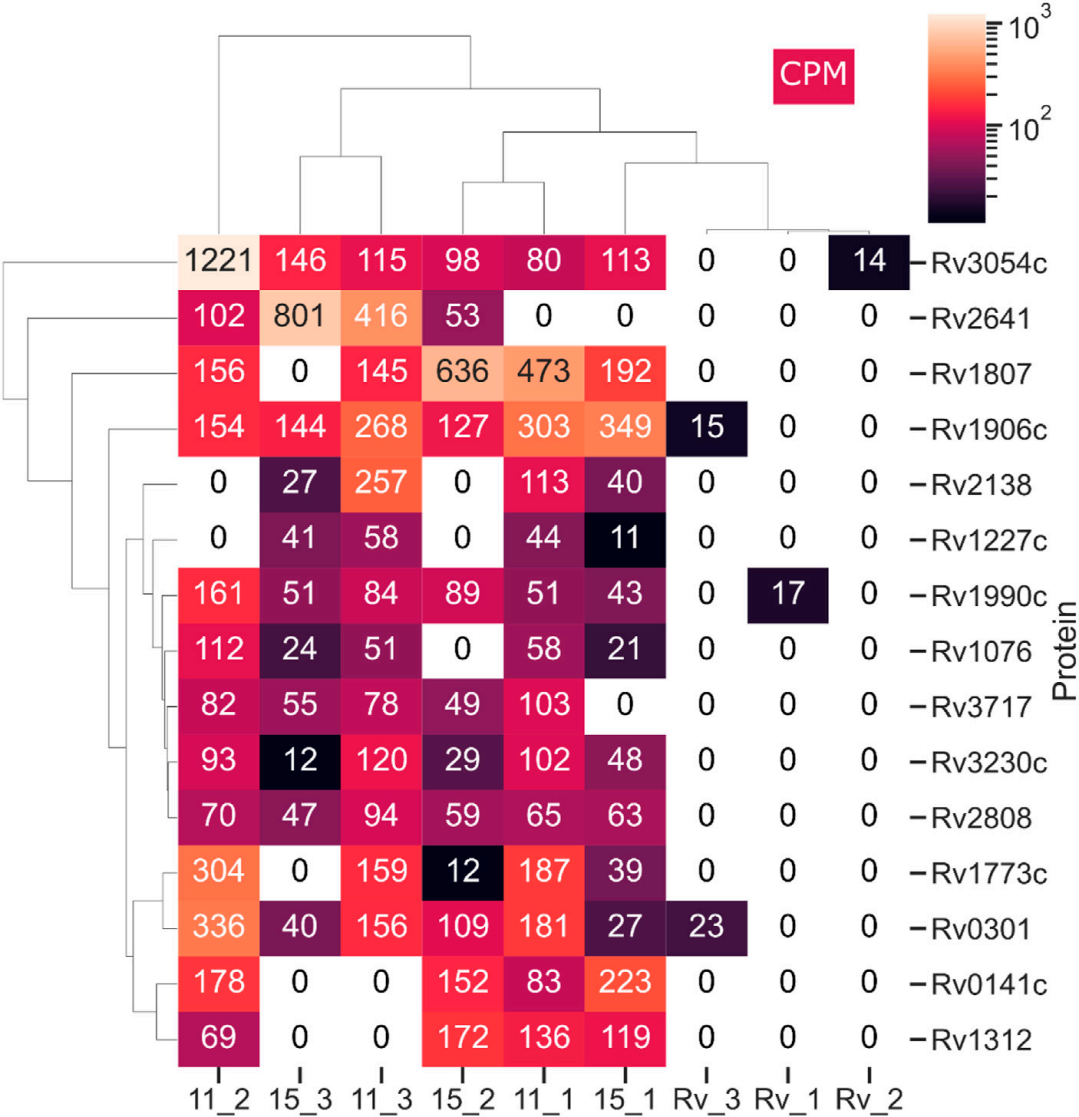
Proteins detectable by LC–MS/MS in M. tuberculosis treated with compounds 11 and 15 but not in the control, untreated cells. This heat map was prepared using the Seaborn package - Python programming. CPM represents counts per million. The total protein was isolated from 3 biological repeats of M. tuberculosis culture supplemented with subinhibitory (0.5 MIC) concentrations of compounds 11 (11_1-3), 15 (15_1-3) and untreated control (Rv_1-3). The isolated protein samples were analyzed on Q Exactive high-performance mass spectrometer.

Subinhibitory concentrations of antibiotics affect bacterial cells and lead to the global modification of their transcriptome and proteome in response to toxic substances. In the set of genes upregulated in the presence of a tested drug, one could identify those that specifically respond to the presence of the drug, not as a response to the growth inhibition *per se*. The monitoring of *M. tuberculosis* gene expression in the presence of isoniazid (INH), performed by DNA microarray, revealed several type II fatty acid synthase enzymes, including AcpM and KasA*,* directly involved in the FAS2 processes inhibited by INH (Wilson et al., 1999). Another group identified efflux, transport, and virulence genes presenting resistance-dependent differences in response to RMP (de Knegt et al., 2013). Waddell SJ et al. (2004) applied a DNA microarray to compare the transcriptional response of tubercle bacilli to six compounds, including INH, isoxyl, and tetrahydrolipstatin. The comparison of profiles allowed the authors to define a set of genes responding to each drug tested and drug-specific changes that may reflect the mode of action of a given drug. Liang Chen et al. (2018) evaluated the changes in DNA methylation and gene expression of bacilli treated with RMP and INH, identifying 68 genes upregulated and 63 genes downregulated by both drugs. To evaluate the global response of *M. tuberculosis* to 2,4-disubstituted pyridine derivatives, we applied LC–MS/MS technology to determine the compound-induced changes directly at the protein level. Tubercle bacilli were induced with subinhibitory concentrations (0.5x MIC) of compounds **11** and **15** for 24 h, and the total proteins were isolated and subjected to analysis as described previously (Brzostek et al., 2021). The comparison of compound-treated samples with the untreated control revealed compound-dependent changes in the proteome of *M. tuberculosis*. We focused our analysis on proteins below the detection level in uninduced culture and enriched significantly in the presence of the tested compounds in all three biological repeats of the experiment. Following treatment of bacilli with compounds **11** and **15,** we identified 53 and 35 proteins, respectively, meeting such criteria (Supplementary Table S4). Fifteen proteins were significantly upregulated in the presence of both chemicals used (Figure 4). The analysis of potential pathways upregulated in the presence of subinhibitory concentrations of compounds **11** and **15** performed using ShinyGO v0.741 software identified PPE family proteins significantly enriched in the presence of compound **11** (Supplementary Table S5). Two of these proteins, Rv1773c and potentially essential Rv1990c (Griffin et al., 2011), were annotated as putative transcriptional regulators. The PPE family is represented by PPE31 (Rv1807); however, PPE32 and PPE33, which are encoded by genes of the same operon, were exclusively induced by compound **11**. PPE31 was reported to be a virulence-associated factor that modulates innate immune responses to mycobacterial infection (Feng et al., 2021). This protein was also detected in the global transcriptional response of *M. tuberculosis* to vancomycin (Provvedi et al., 2009). Compounds **11** and **15** also induced the Rv3230c oxidoreductase; this protein was reported to be a biologically relevant electron transfer partner for membrane-bound stearoyl-CoA delta (9)-desaturase (DesA3), which produces oleic acid, a precursor of mycobacterial membrane phospholipids and triglycerides (Chang Y, 2006). Other proteins overproduced in the presence of both compounds were Rv3717, described as a zinc-dependent amidase able to hydrolyze peptidoglycan (Li et al., 2018), and Rv1076, annotated as esterase/lipase LipU presenting activity for the hydrolysis of short carbon chain substrates (Li et al., 2017). The overproduction in the presence of either compound was also detected for possible toxin VapC2 (Rv0301). VapC toxins are PIN domain endonucleases that in *M. tuberculosis* cleave RNAs essential for decoding at the ribosomal A-site contributing in the persistence process (Winther et al., 2016). The other overproduced proteins were 17-kDa CadI (Rv2641) reported as aq protein induced by cadmium in *M. bovis* and *M. tuberculosis*) (Hotter et al., 2001), and several conserved hypothetical proteins of unknown function (Rv2808, Rv3054c, Rv1906c, Rv0141c, Rv1312, and Rv1227c). Based on our results we are not able to say whether the above proteins are overproduced as a stress response or, some of them, are involved in the cellular processes affected directly by the subinhibitory concentrations of the tested compounds.

## Conclusion

Taking all of the above into account, we found that 2,4-disubstituted pyridine derivatives are effective against intracellularly deposited tubercle bacilli, as well as against *M. tuberculosis* biofilms. The acquired resistance to the investigated compounds occurs at a low efficiency, and selected resistant mutants are not viable in liquid media. The resistance mechanism is related to the upregulation of the efflux pump MmpS5-MmpL5. Subinhibitory concentrations of the investigated compounds induce the expression of various virulence-associated genes.

## Data Availability

The datasets presented in this study can be found in online repositories. The names of the repository/repositories and accession number(s) can be found below: The mass spectrometry proteomics data have been deposited to the ProteomeXchange Consortium via the PRIDE [1] partner repository with the dataset identifier PXD035807. Genomics data is available at BioProject: PRJNA843930.
