# Supplementary material for: 2,4-Disubstituted pyridine derivatives are effective against intracellular and biofilm-forming tubercle bacilli
Source: Front Pharmacol. 2022 Nov 10;13:1004632. doi: 10.3389/fphar.2022.1004632 (PMC9685343; doi:10.3389/fphar.2022.1004632)
Supplement: Supplementary file 2 [file DataSheet1.pdf]

Supplementary materials

2,4-disubstituted pyridine derivatives are effective against intracellular and biofilm-forming tubercle bacilli.

Korycka-Machała M[1], Kawka M[2], Lach J[3], Płocińska R[1], Bekier A[2], Dziadek B[2], Brzostek A[1], Płociński P[1,4], Strapagiel D[3], Szczesio M[5], Gobis K[6], Dziadek J.[1]*

Figure S1. X. BOILED-Egg diagrams for the study compounds.

The white area represents the molecules most likely to be absorbed through the gastrointestinal tract, and the yellow area represents the molecules most likely to permeate to the brain. WLOGP (Y-axis) represents lipophilocity, TPSA (X-axis) represents polarity [Å²].

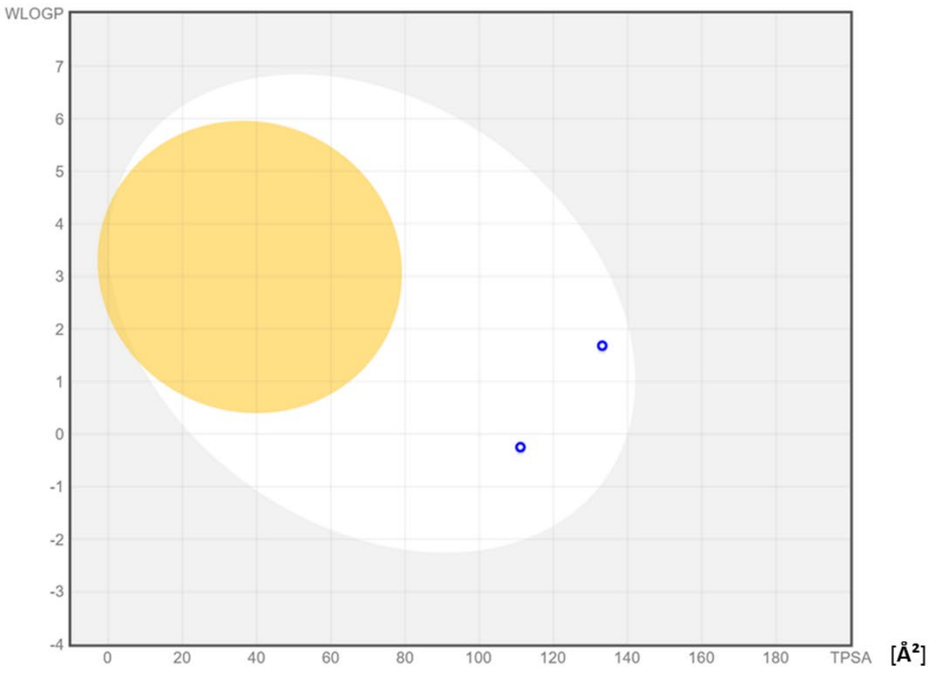

Figure S2. The silencing of mmpR5 transcription using CRISPRi/dCas9 system.

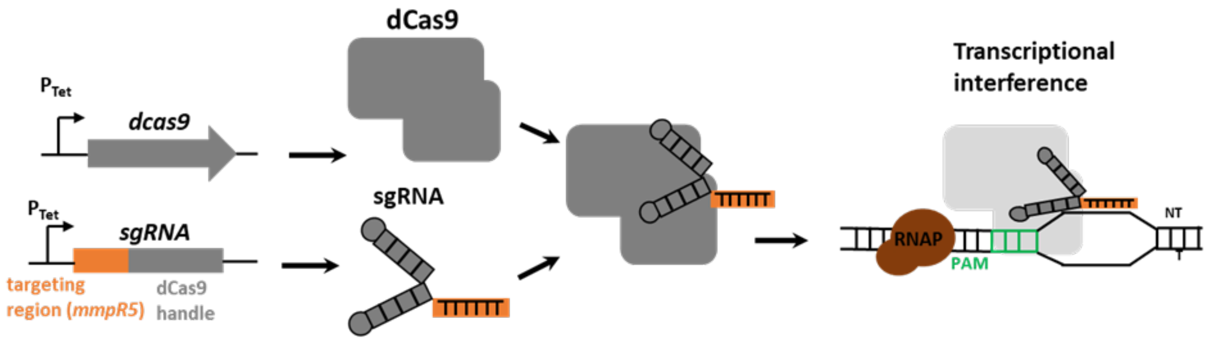

M. tuberculosis strain depleted with MmpR5 was constructed as described in Materials and Methods 2.7. The silencing of *mmpR5* transcription was induced with anhydrotetracycline (aTc, 100 ng/mL). Briefly, *M. tuberculosis* CRISPRi/dCas9^{mmpR5} (*Cas9* ^{mmpR5}) and the control vector carrying an "empty" CRISPRi/dCas9 were grown in 7H9/OADC for 7 days at 37 °C reaching OD_{600} = 0.8-1.0, than refreshed (OD_{600} = 0.1) and induced with aTc (100 ng/µL) for silencing of *mmpR5* transcripts. Next, the culture was refreshed, supplemented with aTc again, and the strains were used to determine MIC for bedaquiline (BDQ) and compound **11**.

Figure S3. Time-dependent viability of *M. tuberculosis* at various concentrations of the compounds **11** (a) and **15** (b).

a

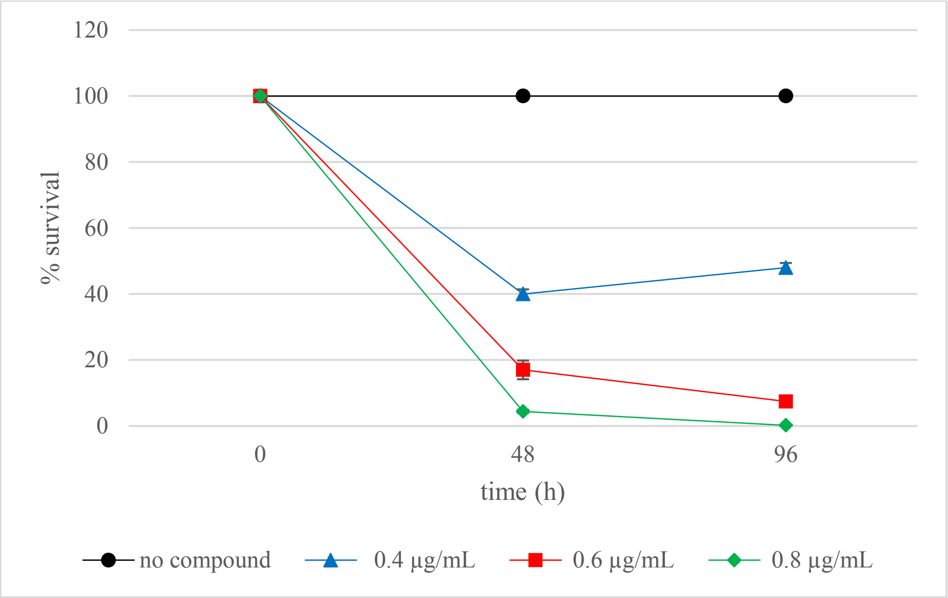

b

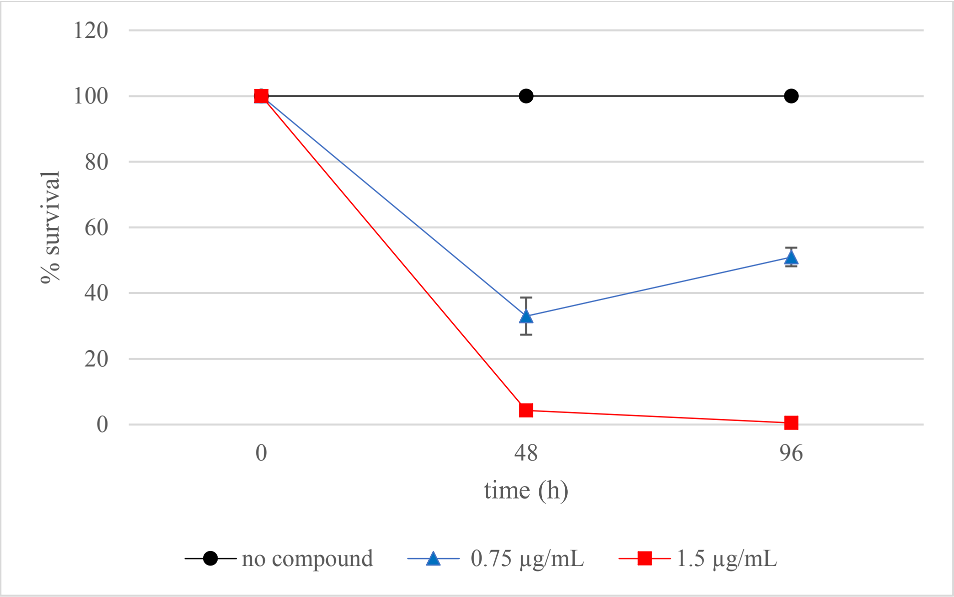

(a) Growth kinetics of the wild-type *M. tuberculosis* strain (black circle) in the presence of 0.4 µg/mL (blue triangle), 0.6 µg/mL (red square) and 0.8 µg/mL (green diamond) of compound **11.** (b) Growth kinetics of the wild-type *M. tuberculosis* strain (black circle) in the presence of 0.75 µg/mL (blue triangle) and 1.5 µg/mL (red square) of compound **15**.
The numbers of viable cells were determined by counting the bacterial colony-forming units (CFUs) on 7H10/OADC plates after 48 and 96 hours. CFU values are the means ± standard deviation from three independent experiments. The graph was prepared using Microsoft Excell/Office 365.

Figure S4. Effects of compounds **11** and **15** on biofilm development by *M. tuberculosis*.

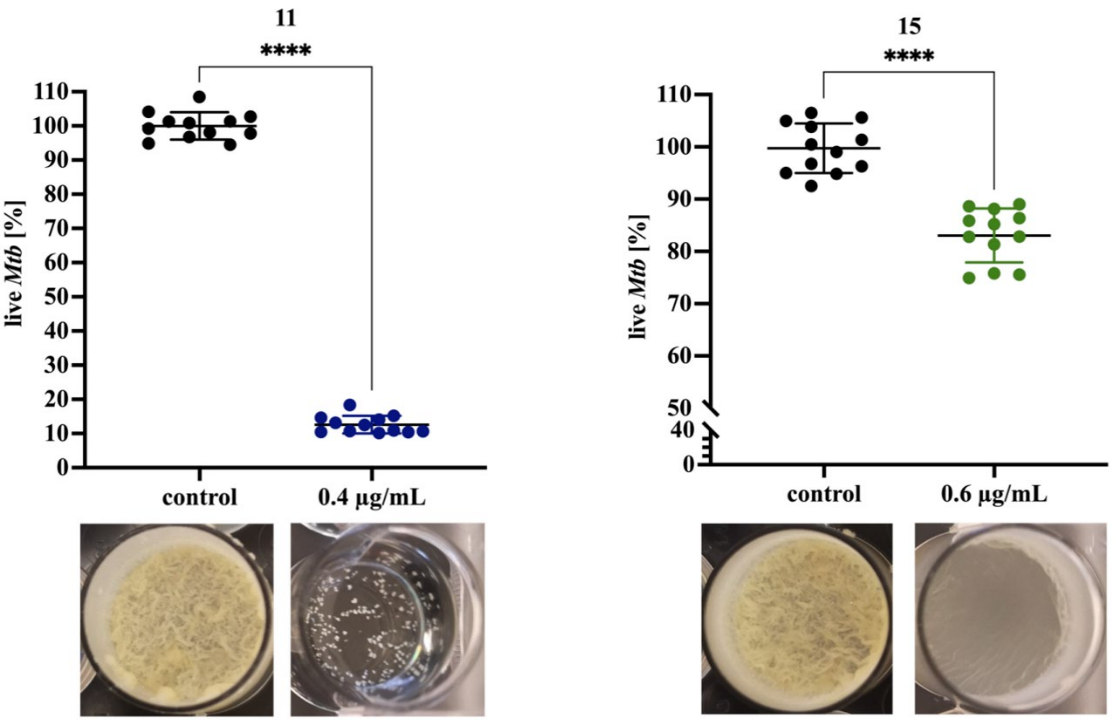

Error bars indicate the standard error of the mean. **** depict the values with significant differences at p<0.001 and p<0.0001, respectively. Data were compared using one-way ANOVA followed by Dunnett's comparisons test. Control represents the no compound wells. Compounds **11** and **15** were used at concentrations of 0.4 (blue dots) and 0.6 (green dots) µg/mL, respectively. The graph was prepared using GraphPad Prism 9 version 9.3.1 (350).

Table S1. The cytotoxicity of compounds **11** and **15** against human macrophages.

Compound	Concentration [µg/mL]	Viability of hMQ [%]
11	0	100
11	0.2	99.3
11	0.4	81
11	0.8	94.2
11	1.0	84.2
**11**	**1.2**	**71.5**
11	2.0	25.2
15	0	100
15	0.6	116.7
15	1.2	97.3
**15**	**1.5**	**68.2**
15	2.4	12.7
15	3.0	10.3
15	15.0	8.6

The viability was determined in duplicates and presented as average.

Table S2. The viability of *M. tuberculosis* in the matured biofilm treated or not with compounds **11** and **15**.

		**11**			**15**		
		**Concentration (µg/mL)**					
**repeats**	**NT**	**0.2**	**0.4**	**0.6**	**0.3**	**0.6**	**1.5**
1	102.2	86.17	85.42	72.56	82.85	81.46	61.63
2	108.13	93.55	82.74	70.29	83.59	78.47	61.36
3	104.76	88.68	82.7	76.41	78.46	77.5	60.54
4	97.76	86.8	66.82	68.31	75.88	70.29	58.38
5	94.16	82.66	69.79	66.1	76.07	68.41	56.48
6	92.99	82.6	68.5	65.66	75.86	68.17	63.87
**average**	**100**	**86.74**	**76**	**69.89**	**78.79**	**74.05**	**60.38**

The viability of the bacilli was determined by fluorescence measurements at an excitation of 550 nm and an emission of 590 nm in the presence of resazurin. NT – not treated.

Table S3. Summary of NGS analysis (see separate data set, Excel)

Table S4. Proteomics, whole data file (see separate data set, Excel)

Table S5. The analysis of proteins enriched in the subinhibitory concentrations of compounds **11** and **15** using ShinyGO v0.741

The only significantly enriched pathway (PPE family, C-terminal) was detected after treatment of *M. tuberculosis* with compound **11**.

Enrichment FDR Compound 11	nGenes	Pathway Genes	Fold Enrichment	Pathways (click for details)
7.4E-03	5	27	14.1	PPE family, C-terminal

N	High level GO category Compound 11	Genes
13	Catalytic activity	lipC vapC2 mftE lipU hisI pgsA2 Rv2181 Rv2190c aroD snoP hflX Rv3230c Rv3717
13	Cellular process	lipC vapC2 mftE hisI pgsA2 tatC Rv2181 aroD snoP rimM Rv3230c mtp Rv3717
12	Metabolic process	lipC vapC2 mftE hisI pgsA2 Rv2181 Rv2190c aroD snoP rimM Rv3230c Rv3717
12	Organic substance metabolic process	lipC vapC2 mftE hisI pgsA2 Rv2181 Rv2190c aroD snoP rimM Rv3230c Rv3717
11	Cellular metabolic process	lipC vapC2 mftE hisI pgsA2 Rv2181 aroD snoP rimM Rv3230c Rv3717
10	Primary metabolic process	lipC vapC2 hisI pgsA2 Rv2181 Rv2190c aroD snoP rimM Rv3230c
9	Binding	vapC2 mftE Rv0880 hisI parD1 hflX rimM Rv3230c Rv3717
9	Hydrolase activity	lipC vapC2 mftE lipU hisI Rv2190c snoP hflX Rv3717

N	High level GO category<br><br>Compound 11	Genes
8	Extracellular region	lipC lipU Rv1910c Rv2190c Rv2576c mpt70 cfp6 espJ
8	Nitrogen compound metabolic process	vapC2 mftE hisI Rv2190c aroD snoP rimM Rv3717
8	Cell periphery	lipC pgsA2 tatC Rv2181 Rv2574 Rv2576c PPE51 Rv3230c
7	Membrane	Rv1312 pgsA2 tatC Rv2181 Rv2574 Rv2576c Rv3230c
6	Intracellular	hisI tatC aroD snoP hflX rimM
6	Biosynthetic process	mftE hisI pgsA2 Rv2181 aroD snoP
6	Ion binding	vapC2 mftE hisI hflX Rv3230c Rv3717
5	Plasma membrane	pgsA2 tatC Rv2181 Rv2574 Rv3230c
5	Intrinsic component of membrane	Rv1312 pgsA2 tatC Rv2181 Rv2576c
3	Regulation of biological process	vapC2 Rv0880 parD1
3	Biological regulation	vapC2 Rv0880 parD1
3	Cellular component organization or biogenesis	rimM mtp Rv3717
3	Catabolic process	lipC snoP Rv3717
3	Pathogenesis	Rv2181 mtp espJ
3	Regulation of metabolic process	vapC2 Rv0880 parD1

N	High level GO category<br><br>Compound 11	Genes
3	External encapsulating structure	lipC Rv2576c PPE51
3	Interspecies interaction between organisms	Rv2181 mtp espJ
3	Regulation of cellular process	vapC2 Rv0880 parD1
3	Organic cyclic compound binding	Rv0880 hflX Rv3230c
3	Heterocyclic compound binding	Rv0880 hflX Rv3230c
2	Protein-containing complex	tatC snoP
2	Cellular component organization	mtp Rv3717
2	Transferase activity	pgsA2 Rv2181
2	Lyase activity	aroD snoP
2	Small molecule binding	hflX Rv3230c
2	Cellular component biogenesis	rimM mtp
2	Protein-containing complex binding	hflX rimM
1	Transporter activity	tatC
1	Electron transfer activity	Rv3230c
1	Biological adhesion	mtp
1	Organelle	rimM

N	High level GO category<br>Compound 11	Genes
1	Response to stimulus	cadI
1	Localization	tatC
1	Molecular function regulator	Rv0880
1	DNA-binding transcription factor activity	Rv0880
1	Protein binding	parD1
1	Extracellular space	mpt70
1	Cell adhesion	mtp
1	Cell surface	lipC
1	Oxidoreductase activity	Rv3230c
1	Transmembrane transporter activity	tatC
1	Envelope	Rv3717
1	Macromolecule localization	tatC
1	Regulation of growth	parD1
1	Response to chemical	cadI
1	Periplasmic space	Rv3717
1	Cell projection	mtp

N	High level GO category<br>Compound 11	Genes
1	Non-membrane-bounded organelle	rimM
1	Intracellular organelle	rimM
1	Positive regulation of biological process	parD1
1	Negative regulation of biological process	vapC2
1	Establishment of localization	tatC
1	Metal cluster binding	Rv3230c
1	Cellular localization	tatC
1	Regulation of molecular function	Rv0880
1	Cell wall organization or biogenesis	Rv3717
1	Carbohydrate derivative binding	hflX
1	Membrane protein complex	tatC

N	High level GO category<br>compound 15	Genes
7	Cellular process	crgA vapC2 pyrF Rv1417 Rv3230c ephA Rv3717
6	Catalytic activity	vapC2 lipU pyrF Rv3230c ephA Rv3717

N	High level GO category<br>compound 15	Genes
6	Metabolic process	vapC2 pyrF Rv1417 Rv3230c ephA Rv3717
6	Membrane	crgA mmpS4 Rv1312 Rv1417 Rv2240c Rv3230c
6	Cellular metabolic process	vapC2 pyrF Rv1417 Rv3230c ephA Rv3717
6	Organic substance metabolic process	vapC2 pyrF Rv1417 Rv3230c ephA Rv3717
5	Intrinsic component of membrane	crgA mmpS4 Rv1312 Rv1417 Rv2240c
4	Binding	vapC2 Rv3230c ephA Rv3717
4	Regulation of biological process	crgA vapC2 vapB29 ephA
4	Biological regulation	crgA vapC2 vapB29 ephA
4	Plasma membrane	crgA mmpS4 Rv1417 Rv3230c
4	Nitrogen compound metabolic process	vapC2 pyrF Rv1417 Rv3717
4	Hydrolase activity	vapC2 lipU ephA Rv3717
4	Ion binding	vapC2 Rv3230c ephA Rv3717
4	Primary metabolic process	vapC2 pyrF Rv3230c ephA
4	Cell periphery	crgA mmpS4 Rv1417 Rv3230c
3	Regulation of metabolic process	vapC2 vapB29 ephA
2	Extracellular region	lipU Rv2240c

N	High level GO category<br><br>compound 15	Genes
2	Response to stimulus	cadI ephA
2	Catabolic process	ephA Rv3717
2	Biosynthetic process	pyrF Rv1417
2	Oxidoreductase activity	Rv3230c ephA
2	Response to chemical	cadI ephA
2	Regulation of cellular process	vapC2 vapB29
2	Regulation of biological quality	crgA ephA
1	Electron transfer activity	Rv3230c
1	Antioxidant activity	ephA
1	Multicellular organismal process	ephA
1	Organelle	ephA
1	Cellular component organization or biogenesis	Rv3717
1	Detoxification	ephA
1	Peroxidase activity	ephA
1	Protein binding	ephA
1	Intracellular	ephA
1	Response to stress	ephA

N	High level GO category<br>compound 15	Genes
1	Pathogenesis	mmpS4
1	Toxic substance binding	ephA
1	Cellular component organization	Rv3717
1	Lyase activity	pyrF
1	Envelope	Rv3717
1	Small molecule binding	Rv3230c
1	Periplasmic space	Rv3717
1	Membrane-bounded organelle	ephA
1	Intracellular organelle	ephA
1	Interspecies interaction between organisms	mmpS4
1	Positive regulation of biological process	ephA
1	Negative regulation of biological process	vapC2
1	Regulation of developmental process	crgA
1	Metal cluster binding	Rv3230c
1	Cellular response to stimulus	ephA
1	Cell wall organization or biogenesis	Rv3717
1	Organic cyclic compound binding	Rv3230c

N	High level GO category<br><br>compound 15	Genes
1	Heterocyclic compound binding	Rv3230c
1	Cellular detoxification	ephA
